# Progress in Medicine

**Published:** 1896-09-05

**Authors:** 


### Medical Progress and Hospital Clinics.

[*The Editor will be glad to receive offers of co-operation and contributions from members of the profession. All letters should be addressed to* The Editor, at the Office, 428, Strand, London, W.C.]

### Progress in Medicine.

### DISEASES OF CHILDREN.

**The Value of Rectal Exploration.**—Dr. George Carpenter[10] points out that many obscure abdominal affections in children are cleared up by means of a rectal examination. In tubercular peritonitis one can make out on bimanual examination a distinct thickening of the peritoneum lining the abdominal wall, or intestinal matting, or thickening of the omentum, or enlarged glands. He also gives instances of the value of this method in connection with cases of horse-shoe kidney, peri-rectal abscess, tumours of the sacrum, intussusception, and affections of the bladder and ovaries. A useful bibliography of the recorded cases of sarcoma of the vagina and of ovariotomy in childhood is appended to his paper.

**Thyroid Treatment in Sporadic Cretinism.**—Drs. Peterson and Bailey[11] have recorded some cases of this affection, and given a synopsis of the published cases. Summarising the results, they find that the results of the thyroid treatment are as beneficial in childhood as in adult life, and the dangers appear to be less, as so far no death has occurred amongst children from this treatment. The physical improvement, as shown by increase in growth, softening of the skin, disappearance of the swellings, eruption of the teeth, &c., has been more marked than the intellectual progress. There has usually been a certain amount of mental improvement, but the authors are inclined to believe that the presence of this affection during the years of development is more serious in its effects than when the disease is delayed until the period of maturity. At the same they think it possible that if treatment were commenced at the first appearance of the disease, and persistently carried out, the natural development of the child might be looked for. Dr. W. B. Noyes[12] has published an interesting study of sporadic cretinism, in which the different views as to the ætiology and pathology of this affection are ably discussed. He records one case of interest. The patient was a child of two years, presenting all the typical features of sporadic cretinism. One quarter of a five grain thyroid tabloid once a day was prescribed, but by mistake a whole tabloid was administered. The result was, that in three days "the tongue and lips had gone down to the natural size, the neck became two inches less in circumference, the swollen abdomen three inches smaller, and the child began to perspire freely, something which she had never been known to do before. The heavy dose of the thyroid extract promptly produced symptoms of poisoning with a temperature of 102 deg. and considerable prostration, and such nervous symptoms as sleeplessness, restlessness, and constant moaning for nearly a week. At the end of this time the entire body desquamated, the dusky, unhealthy skin peeling off, leaving what the mother called a beautiful clear, white, waxy skin. Within two weeks the umbilical hernia disappeared." The dose of the thyroid extract was reduced, and the child continued to make steady improvement.

**Tannigen in Diarrhœa.**—Professor Escherich[13] has found tannigen useful in some forms of diarrhœa in children. Tannigen is a yellowish-grey powder, odourless and tasteless, practically insoluble in water and in dilute acids, but soluble in alkaline solutions and in alcohol. Its beneficial action is explained by the fact that when tannigen comes in contact with alkaline secretions, the tannic acid is liberated and exerts its astringent influence. This makes it specially serviceable in the case of breast-fed infants, in whom the intestinal contents are alkaline; while in artificially-reared children the effect is less marked as the intestinal contents are acid from fermentation. Even in the latter case, however, Dr. Escherich believes that benefit results as the mucous membrane is usually alkaline from the coating of intestinal juices, and at this part, where it is most needed, the tannic acid is set free and acts beneficially. For children under eighteen months the dose is three grains, and for children over that age seven grains, from four to six times a day. In acute inflammatory diseases tannigen is contra-indicated, and the best results are obtained in sub-acute or chronic catarrh of the large intestine. The best test of this condition is the appearance of the stools, which contain much mucus either free or smeared over the fæces.

[10] Ibid. I., 1896, p. 481. [11] Ibid., May 1, 1896. [12] N. Y. Med. Jour., March 14, 1896. [13] Therap. Monatschr., March 9, 1896, and N. Y. Med. Jour., March 28, 1896.

